# Fibroblast Growth Factor 2 Promotes Bladder Hypertrophy Caused by Partial Bladder Outlet Obstruction

**DOI:** 10.3389/fcell.2021.630228

**Published:** 2021-03-30

**Authors:** Ye Gao, Peilin Liu, Fan He, Xingliang Yang, Ronghua Wu, Wei Chen, Longkun Li, Zhenxing Yang

**Affiliations:** Department of Urology, Second Affiliated Hospital, Army Medical University, Chongqing, China

**Keywords:** FGF2, hypertrophy, bladder outlet obstruction, BDNF, urodynamics

## Abstract

Non-invasive biomarkers to identify patients with bladder outlet obstruction (BOO)-related dysfunction are still needed to guide clinical practice. The current study aims to investigate molecular alterations and biomarkers associated with partial BOO (PBOO) in rats. Sprague–Dawley rats were used to establish the BOO model. Serum samples from 60 patients with benign prostatic hyperplasia (BPH) were used for enzyme-linked immunosorbent assay analysis. RNA sequencing and TMT-labeling proteomic analyses were conducted to identify molecular alterations. Masson’s trichrome, H&E, and immunohistochemical staining and western blotting were conducted by using conventional methods following the manufacturer’s instructions. Rats with PBOO experienced hypertrophy of smooth muscle cells and hyperplasia of interstitial cells during the first 4 weeks after the initiation of obstruction. Four weeks later, rats with PBOO showed activation of the adaptive immune response, cell death and apoptosis. The levels of brain-derived neurotrophic factor (BDNF) and fibroblast growth factor 2 (FGF2) in the serum gradually increased in the first 4 weeks and gradually decreased after week 4. FGF2 levels slightly correlated with prostate volume (*R* = 0.156, *P* = 0.0028) but not with age or BMI in BPH patients. No correlations were found between BDNF levels and prostate volume, age or BMI. BOO induces a change from bladder compensation to decompensation at week 4. FGF2 is involved in the development of hypertrophy in the PBOO bladder and shows a positive correlation with prostate volume in BPH patients.

## Introduction

Bladder outlet obstruction (BOO), defined as a high-pressure/low-flow micturition pattern on urodynamic investigation, is a consequence of benign prostatic hyperplasia (BPH) in aging men, bladder neck obstruction (BNO), posterior urethral valve (PUV), and urethral stricture (US) (Metcalfe et al., 2010). An estimated 917 million individuals worldwide experienced lower urinary tract symptoms (LUTS)/BOO in 2008, with the number of affected individuals projected to increase by 9.3% to 1.0 billion in 2013 and by 18.5% to 1.1 billion in 2018 (Irwin et al., 2011). The prevalence of this condition in women has been estimated to be between 2.7 and 29%, and women report fewer classic obstructive symptoms than men (Patel and Nitti, 2001). One of the topical issues from the 2018 International Consultation on Incontinence-Research Society (ICI-RS) meeting shows that functional changes in the bladder occur as a consequence of BOO (Bosch et al., 2019). As Fusco et al. (2018) summarized in their review, bladder wall remodeling due to BOO is hypothesized to involve an initial hypertrophy phase, a subsequent compensation phase and a later decompensation phase.

Bladder outlet obstruction patients with compensation stages show increased detrusor contractility during the voiding phase, often in combination with filling phase detrusor overactivity. As the obstruction continues to progress, patients in the phase of decompensation show detrusor underactivity, and always accompanied by progressive dysuria and larger amount of postvoid residual urine volume (PVR) (Elmissiry et al., 2014). Identifying the function of bladder depend on urodynamics examination, bladder wall thickness (BWT), detrusor wall thickness (DWT) with imaging test, questionnaire of International Prostate Symptom Score (IPSS), et al. However, no international standards or guidelines were made to standardize the diagnosis and we still lack non-invasive techniques or biomarkers to identify which patients are at increased risk of BOO-related bladder dysfunction. Non-invasive markers offer some advantages such as reduced risk of sampling error, objectiveness in the interpretation of the result, appropriateness for repeated measurements and lower cost.

Previous studies have shown that dysregulation of brain-derived neurotrophic factor (BDNF) contributes to the development of stress urinary incontinence (SUI) and bladder pain syndrome/interstitial cystitis (BPS/IC) (Song et al., 2014). Pinto et al. (2010) confirmed that blocking the specific receptor TrkB (high-affinity receptor for BDNF) can significantly improve the symptoms of IC. In addition, Antunes-Lopes et al. (2013) demonstrated that the ratio of BDNF to creatinine in the urine of patients with overactive bladder (OAB) was significantly higher than that in the control group; thus, BDNF is regarded as a potential biological marker for the diagnosis and treatment of OAB. To understand the role of BDNF in the development of BOO, a partial BOO (PBOO) rat model was established using traditional methods (Kitta et al., 2018). In a preliminary experiment, we found that the level of BDNF was higher in rats with PBOO at week 4 and then gradually decreased after week 5. Therefore, the current investigation aimed to clarify the pathophysiology of PBOO and identify biomarkers of the associated process.

## Materials and Methods

### Rats and Establishment of the PBOO Model

Female Sprague–Dawley (SD) rats (age about half a year, *n* = 45, 200–300 g) were obtained from the Experimental Animal Center of Army Medical University (Chongqing, China). All experimental procedures were approved by the Research Council and Animal Care and Use Committee of Army Medical University (approval no. SYXK20070002) and were performed in accordance with the Guide for the Care and Use of Laboratory Animals published by the National Institutes of Health. Briefly, catheterization was achieved by passage of a polyethylene tube through the urethral orifice into the bladder. A 2–3 cm incision was made along the lower abdominal ventral wall, and then the muscle and tissue were dissected until the bladder was exposed. A 3-0 polypropylene suture was used to place a tie around the bladder neck and urethra and then draw out the polyethylene tube. The incision was sutured and disinfected with povidone iodine, and the rat was placed in a warm environment after surgery. The rats in the control group received only a 2–3 cm incision along the lower abdominal ventral wall and were then directly sutured. The whole process was performed under sterile conditions.

### BPH Patients

A total of 60 patients with BPH were recruited from the Second Affiliated Hospital of Army Medical University from 2018 to 2019. All patients were interviewed individually by trained doctors. The patient inclusion criteria were as follows: ① BPH diagnosis made via ultrasonography; ② prostate-specific antigen (PSA) level less than 4.0 ng/ml; and ③ high-pressure/low-flow micturition pattern on urodynamic examination. For the control group, 10 healthy students aged 19–22 years old were recruited from Army Medical University. The control inclusion criteria were as follows: ① absence of LUTS and ② no BPH or LUTS indicated by ultrasonography. This study was conducted with the approval of the ethics committee of Army Medical University (2018-YD-35-01). All participants gave written informed consent.

### Cystometric Evaluation

Female SD rats were subjected to cystometric evaluation 4 weeks after surgery. Inhalation anesthesia was applied by using an R540 Enhanced Small Animal Anesthesia Machine (RWD Life Science Co., Ltd., Shenzhen, China). Polyethylene tubing (Becton, Dickinson and Company, Franklin Lakes, NJ, United States) was inserted into the bladder via an incision in the abdomen. Then, catheters were connected to a three-way valve connected to an infusion pump (AVI 270; 3M, Maplewood, MN, United States) and a pressure transducer (Chengyi Co., Chengdu, China). Cystometric evaluation was performed by infusing warm saline (∼ 37°C) at a rate of 0.2 ml/min. The pressure transducer output was amplified and visualized using a multichannel signal processing system (RM6240C; Chengyi Co.).

### Masson’s Trichrome, Hematoxylin and Eosin (H&E) and Immunohistochemical Staining

Bladder tissues isolated from sacrificed rats were sequentially fixed with 4% paraformaldehyde, dehydrated, and embedded in paraffin. Then, the tissues were deparaffinized and rehydrated. Masson’s trichrome staining (Foot, 1933) and H&E staining (Cardiff et al., 2014) were performed according to a standard protocol. For immunohistochemical (IHC), bladder sections stained with H&E were incubated overnight at 4°C with a primary antibody against TrkB (ab172952, 1:100). The secondary antibody was goat anti-rabbit, goat anti-mouse, or rabbit anti-mouse IgG from a standard SP kit (Zhongshan Co., SPN-9002) and was selected according to the type of primary antibody. A Pannoramic MIDI slice scanner (3D HISTECH) was used to obtain the images.

### Enzyme-Linked Immunosorbent Assay

Enzyme-linked immunosorbent assay (ELISA) kits for rat serum BDNF (SEA011Hu and SEA011Ra) and fibroblast growth factor 2 (FGF2) (SEA551Hu and SEA551Ra) antigen detection were purchased from CLOUD-CLONE Co. (Wuhan, China). The procedure was as follows: (1) a 100 μl serum sample from the rat tail vein was prepared for testing; (2) a 100 μl sample as added to a 96-well plate and incubated at 37°C for 1 h; (3) 100 μl of solution A was added and incubated at 37°C for 1 h; (4) the plate was washed three times; (5) 100 μl of solution B was added and incubated at 37°C for 30 min; (6) the plate was washed five times; (7) 90 μl of TMB substrate was added and incubated at 37°C for 10–20 min; and (8) 50 μl of stop solution was added and read at 450 nm immediately.

### RNA Sequencing Analysis

Bladder tissues were collected from rats in the PBOO and sham groups at week 4 and week 5. Total RNA was extracted using TRIzol^®^ Reagent (Invitrogen, United States) according to the manufacturer’s instructions. High-quality RNA samples were used for library preparation. RNA sequencing (RNA-Seq) libraries were prepared using the Illumina TruSeq^TM^ RNA sample preparation kit (Illumina, San Diego, CA, United States). The libraries were then used for paired-end (PE) sequencing with the Illumina HiSeq4000 platform, and 150 bp PE reads were generated. Quality control of the reads was performed by using SeqPrep^1^ and Sickle^2^ software. Adapter and primer sequences were removed, and sequences with lengths smaller than 20 bp were discarded. After trimming low-quality bases, sequences with quality values less than 10 were discarded. The error rate (%), Q20 and Q30 values, GC content (%), and sequence duplication levels of the resulting high-quality clean reads were then evaluated. The high-quality reads were mapped to the reference, and reference-based assembly of transcripts was performed using Stringtie^3^. Quality control of raw data was conducted with FastQC software^4^. Clean reads were mapped to the reference genome by using HISAT2 v2.0.4. The mapped reads of each sample were assembled by using StringTie v1.3.1 with a reference-based approach. Gene expression values were expressed as reads per kilobase of exon per million fragments mapped (FPKM) using featureCounts software. DEGene analysis was performed using the DESeq2 R package. DESeq2 provides statistical routines for determining differential expression based on gene expression data using a model based on the negative binomial distribution. The resulting *P*-values were adjusted using the Benjamini–Hochberg approach to control the false discovery rate (FDR). Genes with an adjusted *P*-value of <0.05 (FDR < 0.05) by DESeq and a fold change of ≥2 (| log2 fold change| ≥ 1) were considered to be differentially expressed.

### Tandem Mass Tag-Labeling Proteomic Analysis

Bladder tissues were collected from rats in the PBOO and sham groups at week 4 and week 5. The samples were ground and then transferred to a 5-ml centrifuge tube filled with lysis buffer. The supernatant was collected, and the protein concentration was determined with a BCA kit. Then, trypsin digestion was performed. After trypsin digestion, the peptide was desalted on a Strata X C18 SPE column (Phenomenex) and vacuum-dried. The peptide was reconstituted in 0.5 M TEAB and processed according to the manufacturer’s protocol for the tandem mass tag (TMT)/iTRAQ kit. The tryptic peptides were dissolved in 0.1% formic acid and directly loaded onto a homemade reversed-phase analytical column. The gradient was comprised of an increase from 6 to 23% solvent B (0.1% formic acid in 98% acetonitrile) over 26 min, an increase from 23 to 35% in 8 min, an increase to 80% in 3 min and holding at 80% for the last 3 min, all at a constant flow rate of 400 nl/min on an EASY-nLC 1000 UPLC system. The peptides were subjected to NSI followed by tandem mass spectrometry (MS/MS) on an Exactivei^TM^ Plus (Thermo) coupled online to the UPLC. The resulting MS/MS data were processed using the Maxquant search engine (v.1.5.2.8). Tandem mass spectra were searched against the human UniProt database concatenated with the reverse decoy database. Trypsin/P was specified as a cleavage enzyme allowing up to 4 missing cleavages. The FDR was adjusted to <1%, and the minimum score for modified peptides was set to >40.

### Cell Proliferation Assay

The bladder tissues were isolated from the sacrificed rats under aseptic conditions and placed in sterile PBS (phosphate buffered saline) (Gibco, pH 7.4 basic, #lot8119170, China). Using sterile forceps and either small sterile scissors or a sterile scalpel, the bladder tissue was minced to small pieces. The minced bladder tissue was transferred to a 50 ml tube for the following enzymatic digestion. Tissues from rats were digested in a digestion solution containing 2 mg/ml papain (Worthington, #lotLS003120), 100 U/ml Collagenase II (Sigma, CAS No: 9001-12-1, United States), and 100 U/ml Collagenase IV (Sigma, CAS No: 9001-12-1, United States) in DMEM-high glucose medium (Lee et al., 2013). The digested suspension was passed through a 60 μm Steriflip (Millipore, CAS No: SCNY00060), washed twice with washing medium (PBS and 0.04% BSA). The fibroblast and smooth muscle cells were seeded into 6-well plates with DMEM-high glucose medium (Gibco, Life Technologies, Grand Island) and SMCM medium (ScienCell, #lot28299) separately. Recombinant fibroblast growth factor 2 (RPA551Ra01) was purchased from CLOUD-CLONE Co. (Wuhan, China). Cell Counting Kit-8 (CCK-8) reagent (Dojindo, Shanghai, China) were employed to assess cell viability. The Fgf2 group and non-Fgf2 group of fibroblast and smooth muscle cells were cultured in a 96-well plate at the density of 2,000 cells/well and then incubated for 24, 48, 72, 96, 120, and 144 h. A total of 10 μl CCK-8 reagent were added in 100 μl replaced medium before detection. Then, the optical density (OD) values at the wavelength of 450 nm was measured on an enzyme-linked immunosorbent assay plate reader (Varioskan Flash, Thermo Fisher Scientific, Waltham, MA, United States).

### Statistical Analysis

Bladder sc-RNA-seq data were available from the Gene Expression Omnibus (GEO), accession number GSE108097. Fischer Rat RNA-seq data accession number GSE63652. The Gene Set Enrichment Analysis (GSEA) platform was used for pathway analysis. Image-pro plus 6.0 (Media Cybernetics, Inc., Rockville, MD, United States) was employed to measure the area of blue collagen fibers and the percentage area of collagen fibers after Masson staining. 3D panoramic scan software (Scanner model: 3D HISTECH Pannoramic250, Hungary) was used to observe and describe different types of lesions.

The Gene Ontology (GO^5^), Biocarta^6^, and Kyoto Encyclopedia of Genes and Genomes (KEGG^7^) databases were used for pathway analysis. Proteins were classified by GO annotation into three categories: biological process, cellular compartment, and molecular function. For each category, two-tailed Fisher’s exact test was employed to test the enrichment of the differentially expressed protein against all identified proteins. For GO, a corrected *P*-value < 0.05 was considered significant.

## Results

### Establishment of the Rat PBOO Model

**FIGURE 1:**
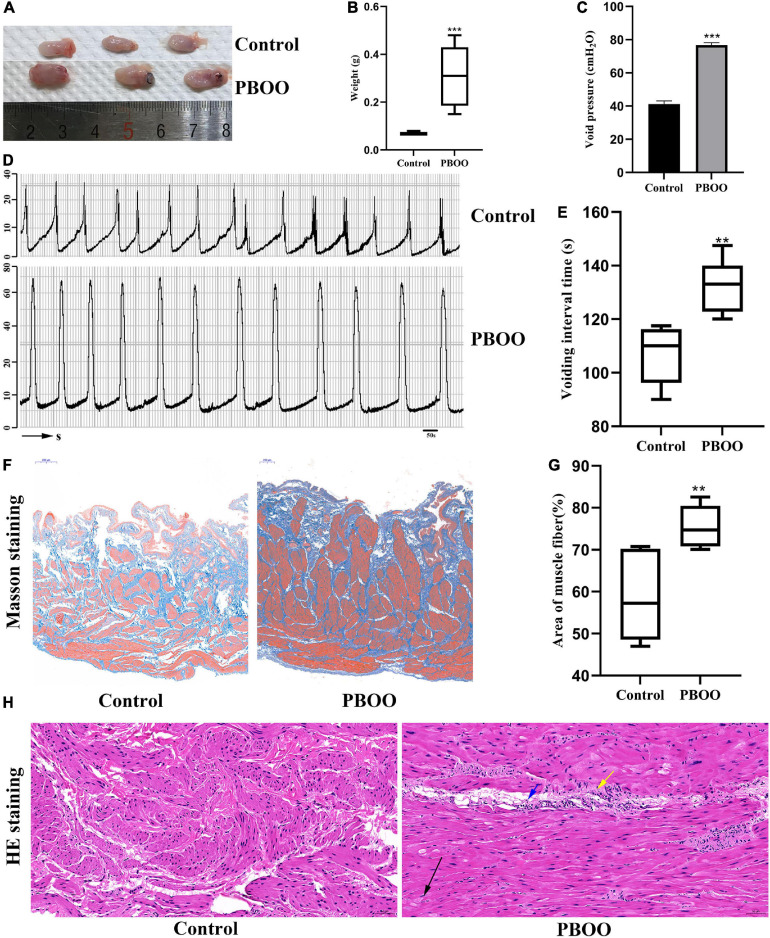
Construction of bladder partial outlet obstruction (PBOO) model in rat. Bladder shape **(A)** was bigger and weight **(B)** was higher in rat of PBOO compared with control group. Cystometric evaluation **(D)** indicated higher voiding pressure **(C)** and lower voiding frequency **(E)** compared with control group. Masson staining of bladder tissue show hypertrophy muscle fiber **(F)** and lower area of collagen fiber **(G)** in PBOO rat. HE staining of bladder tissue demonstrated local muscle fiber swelling **(H)**, cytoplasmic looseness (black arrow), occasional granular mast cells (yellow arrow), and a small number of neutrophils in the blood vessels (blue arrow). *P < 0.05, **P < 0.01, ***P < 0.001.

The PBOO model was successfully established in female rats by using traditional methods (Mattiasson and Uvelius, 1982). Not only bladder shape (Figure 1A) and weight (Figure 1B, *P* < 0.001) but also cystometric measures including voiding pressure (Figures 1C,D) and voiding frequency (Figures 1D,E) differed between the model and control groups. Masson’s trichrome staining indicated thicker muscle fibers (Figure 1F) and a smaller area of collagen fibers (Figure 1G, *P* < 0.01) in rats with PBOO 4 weeks after surgery. H&E staining of bladder tissue demonstrated local muscle fiber swelling, cytoplasmic looseness, occasional granular mast cells, and a small number of neutrophils in the blood vessels (Figure 1H). All characteristics mentioned above indicated that the PBOO rat model was established according to the pathological features of abnormal bladder function caused by obstruction (Sarma and Wei, 2012).

### The Role of Bdnf in the Development of PBOO

**FIGURE 2:**
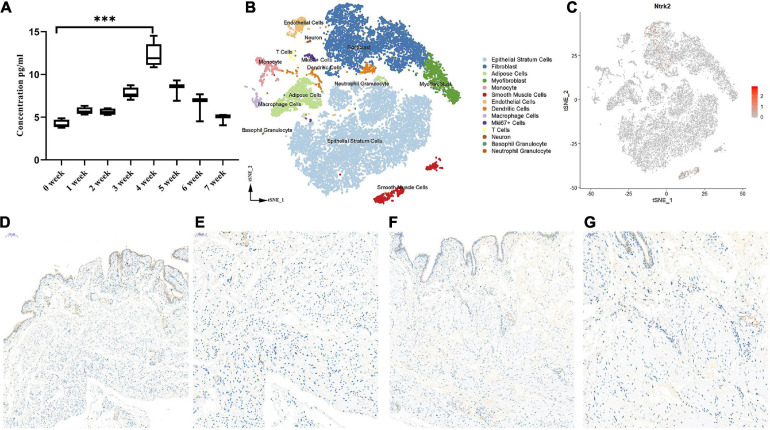
The role of BDNF in the process of PBOO. The level of BDNF in rat serum (*n* = 8, female) gradually increase and reach higher levels on 4 weeks, then gradually decrease 4 weeks later **(A)**. t-SNE analysis of all of the filtered cells (*n* = 19,946) from rat bladder **(B)**. BDNF receptor, Ntrk2 express mainly in interstitial cells and smooth muscle cells (**C**, red color). Immunohistochemistry staining of antibody Trkb2 in normal (**D**, 10× and **E**, 20×) and PBOO (**F**, 10× and **G**, 20×) rat bladder. ***P < 0.001.

To investigate the role of Bdnf in the process of PBOO, serum from the rat tail vein (*n* = 8) was obtained every week. The level of Bdnf antigen detected by ELISA gradually increased, peaked at 4 weeks, and then gradually decreased 4 weeks later (Figure 2A). These results indicated that Bdnf was altered mainly in the first 4 weeks. To clearly elucidate the cell types affected by Bdnf, Ntrk2 (the Bdnf receptor) expression levels were detected in the rat bladders. Combining single-cell sequencing data and IHC staining, we found that Ntrk2 was mainly expressed in interstitial and smooth muscle cells in the bladder (Figures 2B,C). More importantly, no difference in Ntrk2 expression was found in bladder tissue from control or PBOO rats (Figures 2D–G). Therefore, BDNF may act on interstitial and smooth muscle cells of the bladder, and these effects last approximately 4 weeks in rats with PBOO.

### RNA-Seq Analysis of PBOO Bladder Tissues at Week 4

**FIGURE 3:**
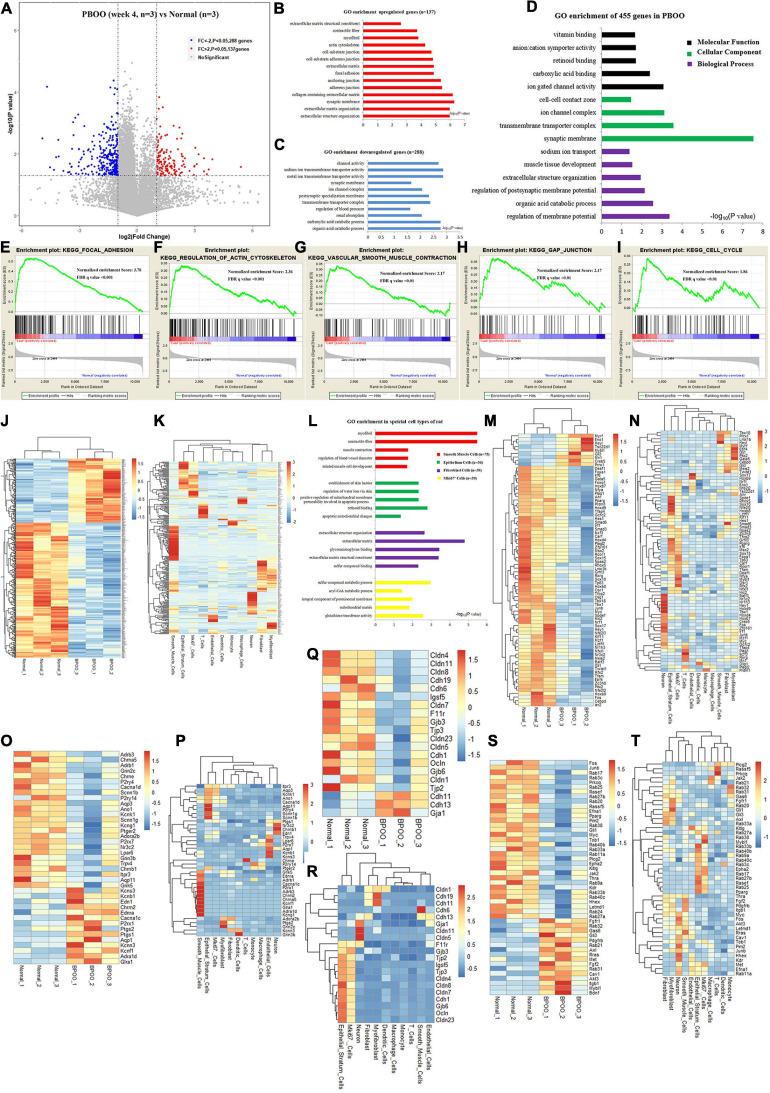
RNA sequencing (RNA-seq) analysis comparing bladder tissues of week 4 PBOO and normal rat. Volcano plot **(A)** show 137 high expression genes (red color) and 288 low expression genes (blue color) in week 4 PBOO rat bladder tissues (*n* = 3) compared with normal group (*n* = 3). GO enrichment analysis of high expression genes (*n* = 137) in PBOO rat bladder tissue **(B)**. GO enrichment analysis of low expression genes (*n* = 288) in PBOO rat bladder tissue **(C)**. GO enrichment analysis of high and low expression genes (*n* = 455) in PBOO rat bladder tissue **(D)**. GSEA analysis show five major KEGG pathway changes in PBOO rat bladder tissues compared normal groups **(E–I)**. Heatmap plot show 137 high expression and 288 low expression genes in two groups **(J)**. Heatmap plot show 455 abnormal expression genes mainly distributed in smooth muscle cells and interstitial cells of rat bladder **(K)**. GO enrichment analysis of abnormal expression genes in different cell type focus on different pathway **(L)**. Picking up 77 high and low expressed transcript factors **(M)** in PBOO rat bladder, and enrichment in different cell types in bladder tissues **(N)**. Picking up 40 high and low expressed ion channel genes **(O)** in PBOO rat bladder, and enrichment in different cell types in bladder tissues **(P)**. Picking up 20 high and low expressed cell connection genes **(Q)** in PBOO rat bladder, and enrichment in different cell types in bladder tissues **(R)**. Picking up 49 high and low expressed growth factors **(S)** in PBOO rat bladder, and enrichment in different cell types in bladder tissues **(T)**.

During the development of the PBOO rat model, a turning point was observed at 4 weeks. To clearly understand the different pathophysiology between PBOO bladder tissues evaluated at week 4 and week 5, RNA-seq and TMT-labeling proteomic analysis were performed on PBOO and control rat bladder tissues. Compared with the control tissues, PBOO bladder tissues at week 4 had 137 genes with increased expression (fold change > 2, and *P* < 0.05) and 288 genes with decreased expression (fold change < −2, and *P* < 0.05) (Figure 3A). The 137 upregulated genes were enriched in pathways related to extracellular matrix structure, contractile fibers, myofibrils, the actin cytoskeleton, etc., which are widely associated with interstitial and smooth muscle cells (Figure 3B). The 288 downregulated genes were mainly enriched in pathways related to channel activity and synaptic membranes (Figure 3C). Overall, the 455 up- and downregulated genes were enriched in pathways related to membrane potential, synaptic membranes and ion gated channel activity (Figure 3D). More importantly, GSEA analysis indicated that five major pathways from the KEGG database (focal adhesion, regulation of actin cytoskeleton, smooth muscle contraction, gap junctions, and cell cycle) were activated in PBOO bladder tissues (Figures 3E–I). To clarify the cell types associated with these up- and downregulated genes, we combined single-cell sequencing (unpublished) and RNA-seq data. The results indicated that the 455 genes with upregulated and downregulated expression were mainly distributed in smooth muscle cells and interstitial cells, which coincided with the GSEA analysis above (Figures 3J,K). Interestingly, genes in different cell types exhibited different pathway activities (Figure 3L). Considering the roles of transcription factors, growth factors, ion channels and cell connections (Supplementary Tables) in the urination function of the bladder, we further analyzed the expression of genes in these subclasses in PBOO bladder tissues. Nine upregulated and 68 downregulated transcription factor genes were identified in PBOO bladder tissues, and these genes were associated with neurons, interstitial cells, epithelial cells, and smooth muscle cells (Figures 3M,N). Fourteen upregulated and 26 downregulated ion channel genes were present in PBOO bladder tissues, and these genes were associated mainly with smooth muscle cells (Figures 3O,P). Three upregulated and 17 downregulated cell connection-related genes were present in PBOO bladder tissues, and these genes were associated mainly with epithelial stratum cells (Figures 3Q,R). 33 high and 16 low expression growth factor related genes were present in PBOO bladder tissues, which associated mainly with smooth muscle cells and epithelial stratum cells (Figures 3S,T).

### RNA-Seq and TMT-Labeling Proteomic Analysis of PBOO Bladder Tissues at Week 5

**FIGURE 4:**
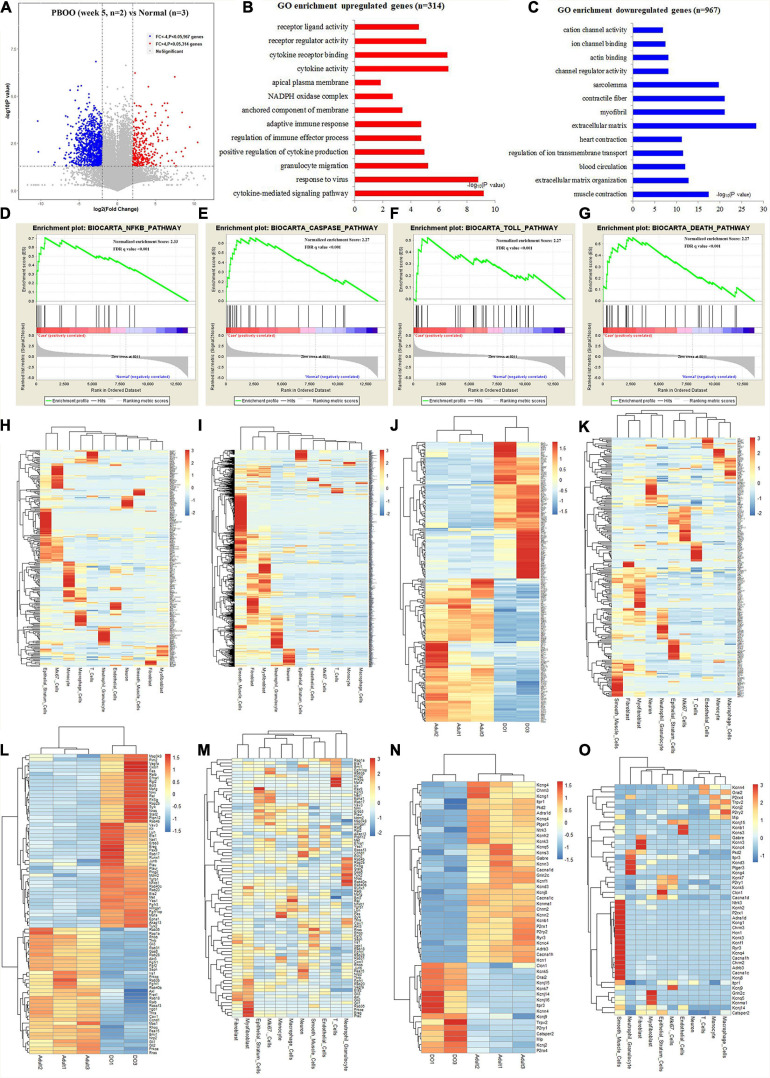
RNA-seq analysis comparing bladder tissues of week 5 PBOO and normal rat. Volcano plot **(A)** show 314 high expression genes (red color) and 967 low expression genes (blue color) in week 5 PBOO rat bladder tissues (*n* = 2) compared with normal (*n* = 3). GO enrichment analysis of high expression genes (*n* = 314) in PBOO rat bladder tissue **(B)**. GO enrichment analysis of low expression genes (*n* = 967) in PBOO rat bladder tissue **(C)**. GSEA analysis show four major biocarta pathway changes in PBOO rat bladder tissues compared normal **(D–G)**. Heatmap plot show 314 high expression gene were distributed in different bladder cell **(H)**. Heatmap plot show 967 low expression gene were distributed in different bladder cell **(I)**. Picking up 250 high and low expressed transcript factors **(J)** in PBOO rat bladder, and enrichment in different cell types in bladder tissues **(K)**. Picking up 83 high and low expressed growth factors **(L)** in PBOO rat bladder, and enrichment in different cell types in bladder tissues **(M)**. Picking up 48 high and low expressed ion channel genes **(N)** in PBOO rat bladder, and enrichment in different cell types in bladder tissues **(O)**.

**FIGURE 5:**
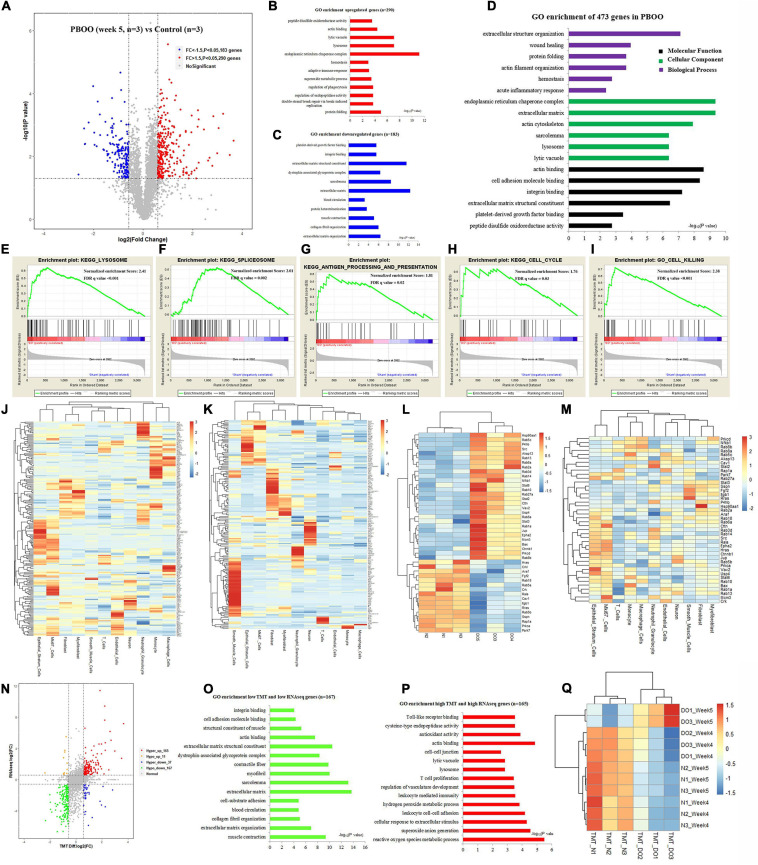
TMT-labeling proteomic analysis comparing bladder tissues of week 5 PBOO and normal rat. Volcano plot **(A)** show 290 high (red color) and 183 low expression proteins (blue color) in week 5 PBOO rat bladder tissues (*n* = 3) compared with normal (*n* = 3). GO enrichment analysis of high expression proteins (*n* = 290) in PBOO rat bladder tissue **(B)**. GO enrichment analysis of low expression proteins (*n* = 183) in PBOO rat bladder tissue **(C)**. GO enrichment analysis of high and low expression proteins (*n* = 473) in PBOO rat bladder tissue **(D)**. GSEA analysis show four major KEGG pathway changes in PBOO rat bladder tissues compared normal **(E–I)**. Heatmap plot show 290 high expression gene were distributed in different bladder cell **(J)**. Heatmap plot show 183 low expression gene were distributed in different bladder cell **(K)**. Picking up 76 high and low expressed growth factors **(L)** in PBOO rat bladder, and enrichment in different cell types in bladder tissues **(M)**. Data consistency between RNA-seq and TMT-labeling proteomic analysis **(N)**. GO enrichment analysis of 167 low expression genes and proteins in PBOO rat bladder tissue **(O)**. GO enrichment analysis of 165 high expression genes and proteins in PBOO rat bladder tissue **(P)**. Pearson correlation between RNA-seq and TMT-labeling proteomic analysis **(Q)**.

However, completely different from the changes in bladder tissues from rats with PBOO at week 4, bladder tissues from rats with PBOO at week 5 showed more abnormal gene expression. In total, 1281 genes, including 314 upregulated (fold change > 4, and *P* < 0.05) and 967 downregulated (fold change < −4, and *P* < 0.05) genes with stricter filtering conditions, were found in bladder tissues from rats with PBOO at week 5 (Figure 4A). A total of 314 upregulated genes were involved in the receptor ligand activity, cytokine activity, and adaptive immune response pathways (Figure 4B). A total of 967 downregulated genes mainly focused on pathways related to cation channel activity, ion channel binding, actin binding, etc. (Figure 4C). GSEA indicated that four pathways from the Biocarta database (Biocarta NF-κB, Biocarta caspase, Biocarta Toll, and Biocarta death) were activated in PBOO bladder tissues (Figures 4D–G). Further analysis indicated that 314 upregulated genes were mainly distributed in epithelial stratum cells, Mki67^+^ cells, and immune cells, including monocytes, macrophages, T cells and neutrophil granulocytes (Figure 4H). A total of 967 genes with downregulated expression were mainly distributed in smooth muscle cells, interstitial cells, and neurons (Figure 4I). The results demonstrated that the immune system was activated in epithelial stratum cells and that cell viability was inhibited in smooth muscle cells, interstitial cells, and neurons in bladder tissues from rats with PBOO at week 5. A total of 123 upregulated and 127 downregulated transcription factor genes were identified in PBOO bladder tissues, and these genes were expressed in all types of cells (Figures 4J,K). Forty-eight upregulated and 35 downregulated growth factor-related genes were present in PBOO bladder tissues (Figures 4L,M). Sixteen upregulated and 32 downregulated ion channel genes were present in PBOO bladder tissues, and these genes were associated mainly with smooth muscle cells (Figures 4N,O). RNA-seq represents changes at only the transcription level, and to validate these changes at the protein translation level, TMT-labeling proteomic analysis was performed in bladder tissues from rats with PBOO at week 5. A total of 290 upregulated (fold change > 1.5, and *P* < 0.05) and 183 downregulated (fold change > −1.5, and *P* < 0.05) proteins were identified in bladder tissues from rats with PBOO compared with control tissues at week 5 (Figure 5A). Similar to the week 5 RNA-seq data, the upregulated proteins were enriched in receptor ligand activity, actin binding, lysosome, etc. (Figure 5B), and the downregulated proteins were enriched in the pathways of platelet-derived growth factor (PDGF) binding, integrin binding, etc. (Figure 5C). Overall, the up- and downregulated proteins were enriched in pathways associated with extracellular structure organization, wound healing, actin binding, cell adhesion molecule binding, etc. (Figure 5D). GSEA indicated that five KEGG pathways (lysosome, spliceosome, antigen processing and presentation, cell cycle, and cell killing) were activated in PBOO bladder tissues (Figures 5E–I). A total of 290 upregulated proteins were mainly distributed in epithelial stratum cells and immune cells (Figure 5J). A total of 183 upregulated proteins were mainly distributed in smooth muscle cells, interstitial cells, and neurons (Figure 5K). Fifty-one upregulated and 25 downregulated growth factor-related proteins were present in PBOO bladder tissues (Figures 5L,M). The consistency of RNA-seq and TMT-labeling proteomic data was evaluated, and 95% of the RNA and protein expression data were synchronized in PBOO bladder tissues at week 5 (Figure 5N); this was also supported by the Pearson correlation test (Figure 5Q). Downregulated (Figure 5O) and upregulated (Figure 5P) genes/proteins were enriched in the pathways mentioned in the previous analysis.

### Fgf2 Expression During the Development of PBOO and Correlation With Clinical Data

**FIGURE 6:**
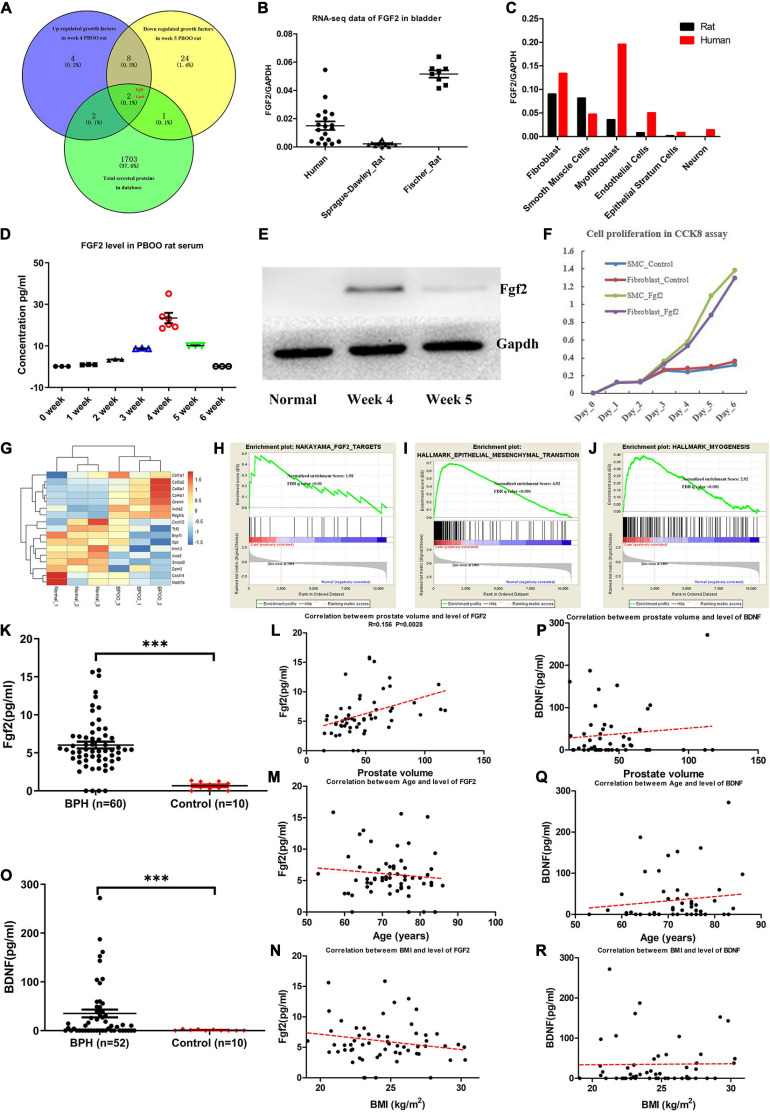
Fgf2 expression in the development of PBOO and correlation with clinical data. Venny plot **(A)** show that Fgf2 and Gas6 were common among high expression in week 4, low expression in week 5 PBOO rat and characteristic for secreted protein. Fgf2 expressed in free-disease bladder tissues **(B)**. Fgf2 expressed in different cell types of bladder tissues **(C)**. The level of Fgf2 in rat serum (*n* = 3, female) from 0∼6 weeks **(D)**. Western blot show Fgf2 expression in normal, week 4 and week 5 PBOO rat bladder tissues **(E)**. Fgf2 promote smooth muscle cell and fibroblasts proliferation in CCK8 assay **(F)**. Heatmap plot show 7 low and 10 high expression gene were distributed between PBOO and normal bladder tissues **(G)**. GSEA analysis show three major pathway changes in PBOO rat bladder tissues compared normal **(H–J)**. BPH patients exhibited higher expression levels of FGF2 **(K)** and BDNF **(O)** in the serum. Pearson correlation between the level of FGF2 in serum of BPH patients and prostate volume **(L)**, age **(M)** and BMI **(N)**. Pearson correlation between the level of BDNF in serum of BPH patients and prostate volume **(P)**, age **(Q)**, and BMI **(R)**. ***p < 0.001.

As reviewed by Fusco et al. (2018), bladder wall remodeling due to BOO is hypothesized to involve an initial hypertrophy phase, a subsequent compensation phase and a later decompensation phase. Consistent with this hypothesis, rats with PBOO at week 4 were in the hypertrophy phase, and rats with PBOO at week 5 were in the compensation phase. We wanted to determine which molecular markers contributed to the development of bladder hypertrophy. Combined analysis of RNA-seq and TMT-labeled proteomic data revealed that Fgf2 contributed mainly to the development of bladder hypertrophy and was also a secreted protein that can be detected in serum (Figure 6A). By analyzing RNA-seq data from published databases, we found that FGF2 expression was nearly undetectable in control bladder tissues in humans, SD rats and Fischer rats (Figure 6B). Regarding cell types, FGF2 was mainly expressed in fibroblasts, smooth muscle cells, and myofibroblasts (Figure 6C). Similarly, the Fgf2 level in serum from rats with PBOO also continuously increased until week 4 and decreased after week 5 (Figure 6D). The western blot results suggest that Fgf2 expression was higher at week 4 and decreased at week 5 and later (Figure 6E). In addition, the CCK8 assay confirmed that Fgf2 can promote the proliferation of fibroblasts and smooth muscle cells *in vitro* (Figure 6F). More importantly, 17 Fgf2 target genes in the Nakayama-FGF2 target pathway were activated in the bladder in rats with PBOO (Figures 6G,H), with activation of the epithelial-mesenchymal transition and myogenesis pathways (Figures 6I,J). FGF2 and BDNF levels in the serum were detected among patients with BPH and normal controls by using ELISA methods. Compared with normal controls, BPH patients exhibited higher expression levels of FGF2 (Figure 6K, *P* < 0.001) and BDNF (Figure 6O, *P* < 0.001) in the serum. In addition, FGF2 levels were slightly correlated with prostate volume (Figure 6L, *R* = 0.156, *P* = 0.0028) but not with age (Figure 6M) or BMI (Figure 6N). No correlation was found between BDNF levels and prostate volume (Figure 6P), age (Figure 6Q) or BMI (Figure 6R). All of the above data indicated that Fgf2 can be regarded as a marker of the compensation phase in the rat PBOO model.

## Discussion

Based on the knowledge that dysregulation of BDNF contributes to the development of LUTS, the current study systematically investigated the pathophysiological process of bladder changes caused by PBOO. A three-stage model was confirmed to simulate BOO-induced bladder remodeling in rats: hypertrophy, compensation and decompensation. Compared with the indicator BDNF, FGF2 can obviously distinguish the stages of hypertrophy and compensation.

Yu et al. (2019) provided single-cell transcriptomic map of human bladders which indicated cell clusters and marker distribution in different cell types. For example, we can easily search marker or activated gene expression in specific type of cells. The accurate location is more convenience for us to understanding the pathophysiology of bladder. To our knowledge, we performed the first RNA-seq and TMT-labeling proteomics analysis to investigate BOO-induced morphological and molecular alterations in the rat bladder. Except for two samples in 5-week PBOO rats, three biological duplicates were done for detecting differential expression in 4-week and 5-week PBOO rats. One sample was excluded because of quality control in the process of library construction. Actually, even two samples in 5-week PBOO rats were used for RNA-seq, our data still convincing. Because the consistence of these two samples shows very high and also, 95% of the RNA and protein expression data were synchronized in PBOO bladder tissues at week 5. For RNA-seq and TMT-labeling proteomics analysis, we conducted differential expression analysis, GO enrichment analysis, GSEA pathway analysis and specific cell type expression based on sc-RNA-seq data analysis. Based on above analysis, we can easily find out high and low expression gene, cell location and related pathways in BOO rat bladder.

From the suggestion of the 2018 ICI-RS meeting (Bosch et al., 2019), the process of BOO development included oxidative stress, upregulation of hypoxia-inducible factor (HIF)-1α and vascular endothelial growth factor (VEGF), increased collagen content and detrusor wall thickening. However, in the rat PBOO model, we found hypertrophy of smooth muscle cells and hyperplasia of interstitial cells at week 4 and activation of the adaptive immune response at week 5. This process was consistent with the stages of hypertrophy and compensation described by Fusco et al. (2018), in a review of available studies on human tissues. However, no evidence showed that rats did not experience oxidative stress because the bladder tissues were extracted and analyzed at only two time points (week 4 and week 5). The life span of mice is shorter than that of humans. Therefore, oxidative stress may occur earlier than week 4. Evidence from available studies demonstrates that BOO induces molecular and morphological alterations in multiple bladder structures, namely, the urothelium, interstitial cells, smooth muscle cells, and neurons (Beppu et al., 2011; Drzewiecki et al., 2012; Iguchi et al., 2014, 2016; Hughes et al., 2016; Michishita et al., 2016). Current data indicated that smooth muscle cells and interstitial cells, including fibroblasts and myofibroblasts, were activated in and before week 4. After week 5, the rats with PBOO exhibited completely different molecular alterations, including activation of the adaptive immune response, inhibition of ion channel activity in smooth muscle cells, and apoptosis of urothelial cells. It seems that the decompensation stage had already appeared in week 5. Therefore, the current study can answer the question of how much time it takes before the bladder decompensates. The results demonstrated that the hypertrophy and compensation stages overlapped with each other. Hypertrophy and hyperplasia to some extent indicate bladder functional compensation.

Fibroblast growth factor 2, also known as bFGF, plays an important role in the development of PBOO, as proven by previous experiments (Imamura et al., 2007; Shi et al., 2009; Negoro et al., 2011). This protein has been implicated in diverse biological processes, such as limb and nervous system development, wound healing, and tumor growth (Chen et al., 2004). In the current analysis, FGF2 expression gradually increased after obstruction began, reached a peak level in week 4 and then gradually decreased after week 5. In control bladder tissues of rats and humans, no FGF2 was detected in bladder cells or in the serum. In addition, we found that the FGF2 level was slightly positively correlated with prostate volume, which may indicate that FGF2 is an important marker for severe disease in BPH patients. Zhu et al. (2008) noted a steady and significant increase in bFGF mRNA expression with the aggravation of BOO in Wistar rats. More importantly, a significant negative correlation was demonstrated between detrusor contraction force and the bFGF mRNA level (Zhu et al., 2008). However, the authors observed changes among only the control group, the BOO week 2 group and the BOO week 6 group, which did not reflect a dynamic process. Imamura et al. (2009) found that bFGF induces hypersensitivity to a cholinergic stimulus in the bladder via altered expression of connexin 43 in rats with BOO at week 4. However, in the current investigation, cell connexin proteins tended to be suppressed in the process of SMC hypertrophy. In summary, the role of FGF2 in the development of BOO was established, and further studies should focus on the factors driving FGF2 expression.

Some limitations of the present study should be mentioned. First, we still need more data regarding the development of PBOO in rats after week 5. Second, a limited number of patients were enrolled in the current clinical investigation. Also, the source of FGF2 in patient serum was obscured. FGF2 is a secreted protein which was not specifically expressed in bladder, also possibly come from tissues of hyperplasia prostate, kidney, and liver, et al. (Steringer and Nickel, 2018). More studies regarding the correlation between FGF2 level and parameters of bladder hypertrophy, such as BWT, DWT, even PVR need to be performed. Last, the molecular mechanism of FGF2 in bladder remodeling associated with PBOO remains unclear, and it remains difficult to target a single signaling pathway due to broad downstream effects.

## Conclusion

In conclusion, BOO induced substantial morphological and molecular alterations in the rat bladder between week 4 and week 5. The turning point from compensation to decompensation occurred at week 4 in rats with PBOO. FGF2 is involved in the development of hypertrophy in the PBOO bladder and shows a positive correlation with prostate volume in BPH patients.

## Data Availability Statement

The GEO accession number for the RNA-seq data is GSE167430. The mass spectrometry proteomics data have been deposited to the ProteomeXchange Consortium *via* the PRIDE partner repository (http://www.ebi.ac.uk/pride) with the dataset identifier PXD024378.

## Ethics Statement

The studies involving human participants were reviewed and approved by the Ethics Committee of Army Medical University (2018-YD-35-01). The patients/participants provided their written informed consent to participate in this study. The animal study was reviewed and approved by Research Council and Animal Care and Use Committee of Army Medical University (approval no. SYXK20070002).

## Author Contributions

ZY and LL conceived, designed, supervised the project, wrote the manuscript, and responsible for all data present in current research. FH, XY, and RW were responsible for the BPH sample collection. YG and PL performed the experiments. ZY and WC conducted the bioinformatics analysis. All authors contributed to the article and approved the submitted version.

## Conflict of Interest

The authors declare that the research was conducted in the absence of any commercial or financial relationships that could be construed as a potential conflict of interest.

## Footnotes

1 https://github.com/jstjohn/SeqPrep

2 https://github.com/najoshi/sickle

3 http://ccb.jhu.edu/software/stringtie/

4 http://www.bioinformatics.babraham.ac.uk/projects/fastqc/

5 http://www.geneontology.org

6 https://cgap.nci.nih.gov/Pathways/BioCarta_Pathways

7 http://www.genome.jp/kegg/
